# Supplementary material for: Neonatal Vitamin D and Associations with Longitudinal Changes of Eczema up to 25 Years of Age
Source: Nutrients. 2024 Apr 26;16(9):1303. doi: 10.3390/nu16091303 (PMC11085504; doi:10.3390/nu16091303)
Supplement: Supplementary file 1 [file nutrients-16-01303-s001.zip › nutrients-2931097-supplementary.pdf]

Supplementary Materials

Figures

Figure S1 Directed acyclic graph (DAG) for Model 1

Figure S2 Directed acyclic graph (DAG) for Model 2

Figure S3 Eczema phenotype from birth to 25 years old in the MACS cohort (Four classes)

Tables

Table S1 The selection of the best-fitting model from latent class analyses

Table S2 Comparison of basic characteristics of participants with and without neonatal vitamin D measurement

Table S3 The distribution of vitamin D by seasons

Table S4 The association between neonatal vitamin D and prevalent eczema from birth to 25 years of age stratified by maternal prenatal smoking

Table S5 The associations between neonatal vitamin D and longitudinal eczema phenotype from birth to 12 years of age stratified by mother's birthplace

Table S6 The associations between neonatal vitamin D and longitudinal eczema phenotype from birth to 25 years of age stratified by mother's birthplace

Table S7 The associations between neonatal vitamin D and longitudinal eczema phenotype from birth to 25 years of age stratified by maternal smoking during pregnancy

Table S8 The associations between neonatal vitamin D and longitudinal eczema phenotype from birth to 25 years of age stratified by paternal education level

Table S9 The association between neonatal vitamin D and eczema/sensitisation phenotype

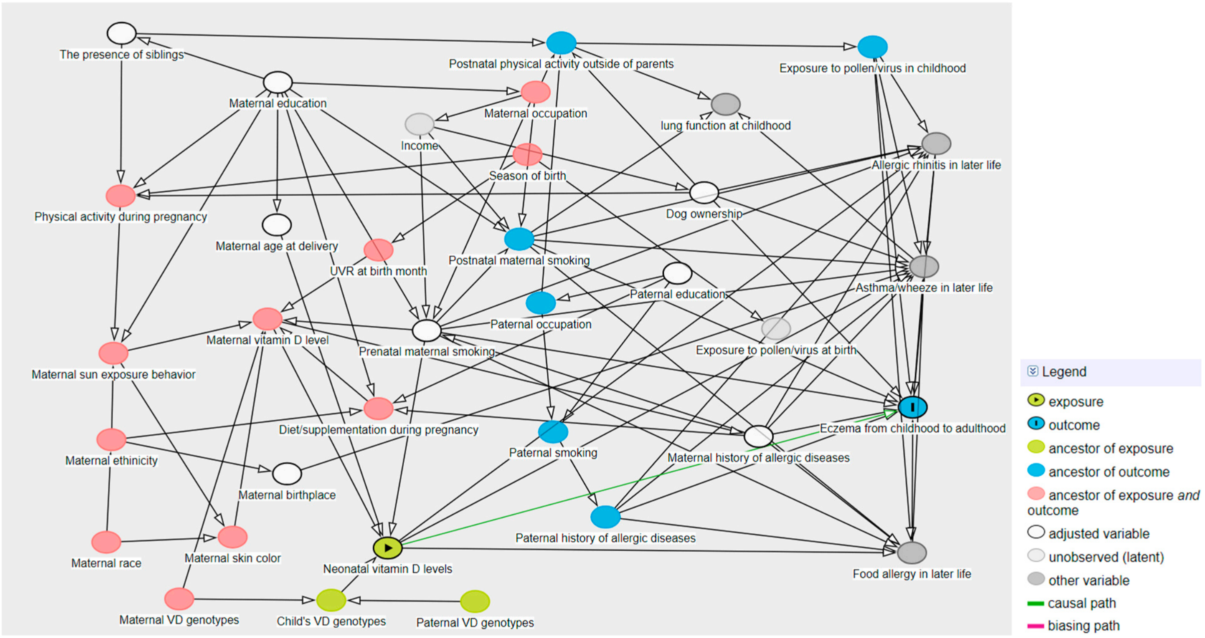

Figure S1 Directed acyclic graph (DAG) for model 1. The exposure is neonatal vitamin D levels and the outcome in this study focused on eczema from childhood to early adulthood. The minimum set of adjustable variables was dog ownership (having dog vs. no dogs), maternal eczema history, mother's age when delivery, maternal smoking during pregnancy, the presence of any siblings, maternal birthplace, maternal and paternal education, as they may impact both neonatal vitamin D levels and eczema outcomes in children.

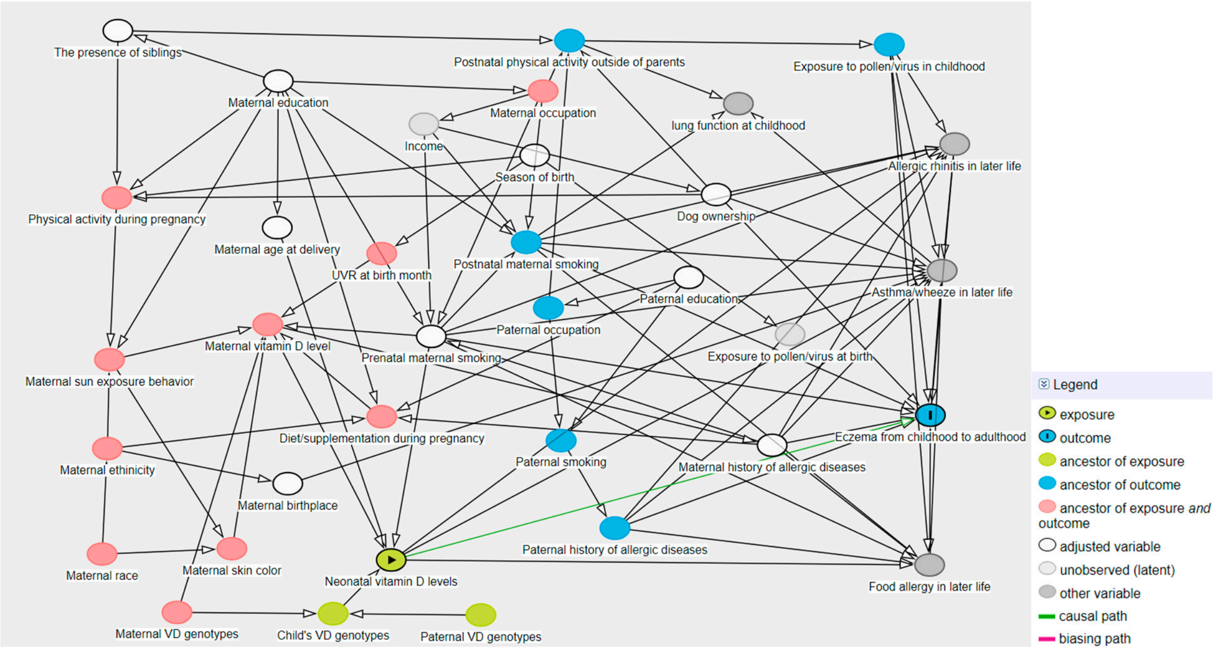

Figure S2 Directed acyclic graph (contested DAG) for Model 2. Adjustable variables are all the adjusted variables in Figure S1 and the **season of birth**.

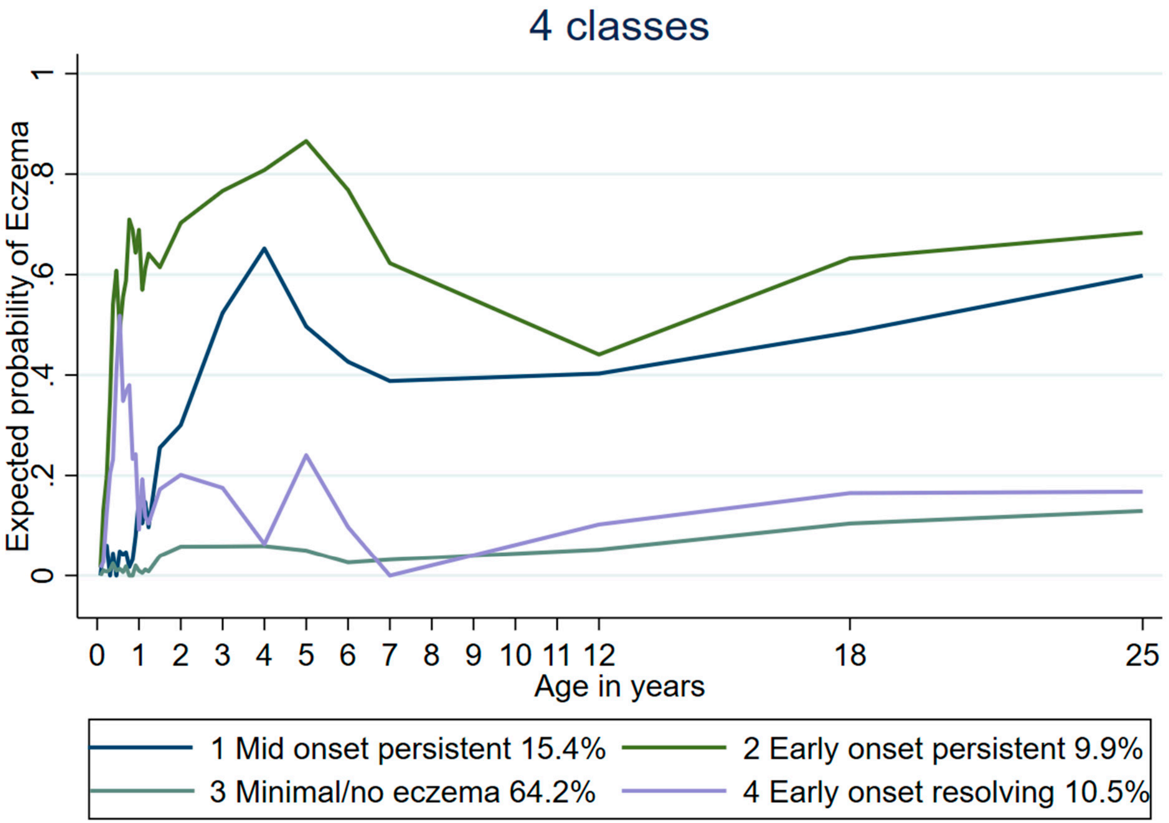

Figure S3 Eczema phenotype from birth to 25 years old in the MACS cohort (Four classes)

Table S1 The selection of the best-fitting model from latent class analyses

**Classes Number**	**Entropy value**	**Smallest class portion**	**AIC**	**BIC**
2 classes	0.94	0.18	7998	8233
3 classes	0.82	0.15	7790	8140
4 classes	**0.87**	**0.10**	7620	**8067**
5 classes	0.86	0.08	7577	8091
6 classes	0.78	0.06	7561	8150

Table S2 Comparison of basic characteristics of participants with (224) and without (396) neonatal vitamin D measurement

Characteristics	Sample without a measurement (n=396)	Sample with measurement (n=224[†])	P value
**Baseline**			
Maternal age (y), mean±SD	30.9±4.4	31.8±4.3	0.026
Maternal birthplace			0.688
Australia/New Zealand	86.4	87.5	
Others	13.6	12.5	
Dog ownership (%)			0.147
Yes	32.6	26.8	
No	65.7	69.6	
Unknown	1.7	3.6	
Maternal eczema-Yes (%)	41.0	35.3	0.159
Maternal food allergy -Yes (%)	38.4	38.8	0.911
Maternal hay fever-Yes (%)	61.0	59.8	0.771
Maternal asthma-Yes (%)	44.8	40.6	0.313
Paternal eczema -Yes (%)	19.2	22.8	0.289
Paternal food allergy -Yes (%)	21.5	21.0	0.884
Paternal hay fever-Yes (%)	45.5	47.3	0.667
Paternal asthma-Yes (%)	26.6	24.1	0.496
Maternal educational level (%)			0.097
Primary school or less	0.3	0	
Secondary school	43.9	35.7	
University degree or higher	55.8	64.3	
Paternal educational level (%)			<0.001
Secondary school	44.8	28.1	
University degree or higher	55.2	71.9	
Smoking during pregnancy (%)			0.007
Ever	28.6	18.8	
Never	71.4	81.2	
Older siblings (%)			0.134
0	37.6	44.7	
1-2	54.8	46.4	

≥3	7.6	8.9	
Sex of newborn (%)			0.928
Female	49.3	49.8	
Male	50.7	50.2	
Birthweight (g), mean±SD	3469±525	3467±509	0.984
Season of birth (%)			0.437
Spring	29.6	26.3	
Summer	21.2	19.2	
Autumn	21.2	26.8	
Winter	28.0	27.7	
**Eczema age**	**Eczema prevalence in samples without vitamin D measures (n/N)**	**Eczema prevalence in samples with vitamin D measures (n/N)**	**P value**
1-year	148/395 (37.47%)	70/224 (31.25%)	0.120
2-years	115/373 (30.83%)	64/222 (28.83%)	0.607
6-years	37/266 (13.91%)	26/176 (14.77%)	0.799
12-years	29/204 (14.22%)	25/157 (15.92%)	0.652
18-years	42/209 (20.10%)	47/198 (23.74%)	0.374
25-years	39/143 (27.27%)	32/123 (26.02%)	0.817

† There was one observation with the 25(OH)D value as 0 (ID=83) that was also included in the table.

‡ Chi-squared tests were used to test the difference between the groups with/without vitamin D measures.

Table S3 The distribution of 25(OH)D3 by the season of birth

Season of birth	Mean (sd, nmol/L)	Median (nmol/L)	P25 (nmol/L)	P75 (nmol/L)	Total (n=)
**Spring** (September to November)	31.4 (16.4)	28.6	20.9	39.8	58
**Summer** (December to February)	47.5 (19.5)	45.1	32.9	60.6	43
**Autumn** (March to May)	42.1 (19.4)	39.3	28.5	53.3	60
**Winter** (June to August)	26.5 (12.7)	22.9	16.9	34.4	62
Total	35.7 (18.7)	32.5	21.6	44.7	223

Table S4 The association between neonatal vitamin D and prevalent eczema from birth to 25 years of age stratified by maternal prenatal smoking

Eczema age	Ever maternal smoking (n=42)		Never maternal smoking (n=181)		p-value of the interaction with the mother smoking
	OR (95%CI) †	p-value	OR (95%CI) †	p-value	
0-1y (n/N‡=70/220)	0.99 (0.84-1.19)	0.990	1.46 (0.95-2.23)	0.083	**0.11**
1-2y (n/N=63/214)	**0.78 (0.63-0.96)**	**0.021**	1.33 (0.85-2.08)	0.214	**0.04**
6-y (n/N=26/174)	0.86 (0.65-1.13)	0.280	0.93 (0.50-1.72)	0.807	0.82
12-y (n/N=25/155)	0.83 (0.61-1.13)	0.245	1.26 (0.72-2.22)	0.424	0.22
18-y (n/N=47/196)	1.09 (0.88-1.34)	0.427	0.77 (0.46-1.32)	0.349	0.25
25-y (n/N=32/122)	0.93 (0.72-1.20)	0.566	1.04 (0.54-2.01)	0.898	0.74
1y-25y (n=222)	0.92 (0.81-1.05)	0.205	1.12 (0.84-1.48)	0.456	**0.09**

† This analysis model was adjusted for dog ownership, maternal eczema history, mother's age when delivery, mother's birthplace, maternal education, and paternal education.

‡ N: The total number of participants with vitamin D and eczema outcomes. n: of these, the number who had eczema.

Table S5 The associations between neonatal vitamin D and longitudinal eczema phenotype from birth to 12 years of age stratified by mother's birthplace

Eczema subclasses	Mother born in Australia/New Zealand(n=195)		Mother born out of Australia/New Zealand(n=28)		P-value of interaction with mother's birthplace
	aMOR (95%CI) §	p-value	aMOR (95%CI) §	p-value	
Early-onset-persistent (n/N‡=22/223)	0.82 (0.63-1.07)	0.151	**0.27 (0.11-0.68)**	**0.005**	**0.02**
Early-onset-resolving (n/N=18/223)	1.33 (1.06-1.68)	0.014	1.04 (0.59-1.83)	0.880	0.43
Mid-onset-persistent (n/N=25/223)	0.96 (0.76-1.20)	0.693	1.07 (0.63-1.82)	0.794	0.69
Mid-onset-resolving (n/N=4/223)	0.80 (0.45-1.41)	0.441	**0.02 (0.001-0.56)**	**0.020**	**0.05**
Minimal/no eczema (n/N=154/223)	1.00 (Reference)	-	1.00 (Reference)	-	Reference

† The analysis was Adjusted for the mother's age when gave birth, the presence of siblings, maternal prenatal smoking, dog ownership, maternal eczema history, maternal education, and paternal education.

‡ N: The total number of participants with vitamin D and eczema outcomes. n: of these, the number in each eczema subclass.

§ aMOR: Adjusted multinominal OR.

Table S6 The associations between neonatal vitamin D and longitudinal eczema phenotype from birth to 25 years of age stratified by mother's birthplace

**Eczema subclasses**	**Born in Australia/New Zealand (n=195)**		**Born outside Australia/New Zealand (n=28)**		**Interaction P-value**
	**aMOR (95%CI)** [§]	**p-value**	**aMOR (95%CI)** [§]	**p-value**	
Early-onset-persistent (n/N[‡]=21/223)	0.84 (0.64-1.10)	0.206	**0.33 (0.15-0.71)**	**0.004**	**0.021**
Early-onset-resolving (n/N=20/223)	1.23 (0.98-1.55)	0.075	0.70 (0.27-1.79)	0.452	0.246
Mid-onset-persistent (n/N=31/223)	1.01 (0.81-1.26)	0.918	1.12 (0.66-1.91)	0.670	0.730
Minimal/no eczema (n/N=151/223)	-	-	-	-	-

[†] The analysis was Adjusted for the mother's age when gave birth, the presence of siblings, maternal prenatal smoking, dog ownership, maternal eczema history, maternal education, and paternal education.

[‡] N: The total number of participants with vitamin D and eczema outcomes. n: of these, the number in each eczema subclass.

[§] aMOR: Adjusted multinominal OR.

Table S7 The associations between neonatal vitamin D and longitudinal eczema phenotype from birth to 25 years of age stratified by maternal smoking during pregnancy

**Eczema subclasses**	**Ever maternal smoking (n=42)**		**Never maternal smoking (n=181)**		**Interaction P-value**
	**aMOR (95%CI)** [§]	**p-value**	**aMOR (95%CI)** [§]	**p-value**	
Early-onset-persistent (n[‡]=21)	0.36 (0.80-1.64)	0.189	0.77 (0.59-1.02)	0.065	0.332
Early-onset-resolving (n=20)	1.67 (1.08-2.59)	0.021	1.15 (0.88-1.49)	0.314	0.147
Mid-onset-persistent (n=31)	**1.43 (1.01-2.04)**	**0.046**	0.97 (0.76-1.24)	0.812	**0.079**
Minimal/no eczema (n=151)	-	-	-	-	-

[†] The analysis was Adjusted for the mother's age when gave birth, the presence of siblings, maternal prenatal smoking, dog ownership, maternal eczema history, maternal education, and paternal education.

[‡] N: The total number of participants with vitamin D and eczema outcomes. n: of these, the number in each eczema subclass.

[§] aMOR: Adjusted multinominal OR.

Table S8 The associations between neonatal vitamin D and longitudinal eczema phenotype from birth to 25 years of age stratified by paternal education level

**Eczema subclasses**	**Paternal education>=Y15 (n=161)**		**Paternal education<Y15 (n=62)**		**Interaction P-value**
	**aMOR (95%CI)** [§]	**p-value**	**aMOR (95%CI)** [§]	**p-value**	
Early-onset-persistent (n/N[‡]=21/223)	0.95 (0.73-1.23)	0.680	**1.43 (1.01-2.03)**	**0.045**	**0.062**
Early-onset-resolving (n/N=20/223)	0.82 (0.64-1.04)	0.106	0.32 (0.40-2.27)	0.245	0.338
Mid-onset-persistent (n/N=31/223)	1.16 (0.90-1.50)	0.245	1.26 (0.66-2.41)	0.484	0.824
Minimal/no eczema (n/N=151/223)	-	-	-	-	-

[†] The analysis was Adjusted for the mother's age when gave birth, the presence of siblings, maternal prenatal smoking, dog ownership, maternal eczema history, maternal education, and paternal education.

[‡] N: The total number of participants with vitamin D and eczema outcomes. n: of these, the number in each eczema subclass.

[§] aMOR: Adjusted multinominal OR.

Table S9 The association between neonatal vitamin D and eczema/sensitisation phenotype

Age	Eczema/sensitisation subclasses	Unadjusted Model		Model 1 †		N‡
		MOR (95%CI) §	p-value	aMOR (95%CI) §	p-value	
6 months	Atopic eczema (n=12)	0.99 (0.71-1.37)	0.930	0.86 (0.60-1.24)	0.426	203
	Non-atopic eczema (n=34)	1.09 (0.90-1.33)	0.366	1.13 (0.92-1.39)	0.229	
	Asymptomatic sensitized (n=30)	1.11 (0.91-1.36)	0.292	1.11 (0.89-1.38)	0.354	
	Asymptomatic (n=127)	Reference group	-	Reference group	-	
12 months	Atopic eczema (n=27)	1.02 (0.82-1.27)	0.884	0.97 (0.77-1.23)	0.819	209
	Non-atopic eczema (n=39)	1.03 (0.85-1.24)	0.795	1.04 (0.85-1.27)	0.675	
	Asymptomatic sensitized (n=32)	0.87 (0.68-1.10)	0.229	0.84 (0.65-1.09)	0.195	
	Asymptomatic (n=111)	Reference group	-	Reference group	-	
2 years	Atopic eczema (n=21)	0.88 (0.65-1.18)	0.382	0.86 (0.64-1.17)	0.342	179
	Non-atopic eczema (n=27)	0.92 (0.71-1.18)	0.503	0.93 (0.69-1.24)	0.608	
	Asymptomatic sensitized (n=40)	1.14 (0.94-1.38)	0.187	1.17 (0.95-1.44)	0.148	
	Asymptomatic (n=91)	Reference group	-	Reference group	-	
12 years	Atopic eczema (n=15)	0.89 (0.64-1.23)	0.465	0.90 (0.64-1.26)	0.534	153
	Non-atopic eczema (n=10)	0.84 (0.56-1.26)	0.399	0.86 (0.57-1.30)	0.470	
	Asymptomatic sensitized (n=75)	0.96 (0.80-1.16)	0.695	0.97 (0.79-1.19)	0.794	
	Asymptomatic (n=53)	Reference group	-	Reference group	-	
18 years	Atopic eczema (n=34)	1.01 (0.80-1.29)	0.923	1.05 (0.81-1.34)	0.726	179
	Non-atopic eczema (n=8)	1.21 (0.85-1.73)	0.281	1.23 (0.83-1.84)	0.301	
	Asymptomatic sensitized (n=86)	1.01 (0.84-1.23)	0.881	1.02 (0.83-1.25)	0.842	
	Asymptomatic (n=51)	Reference group	-	Reference group	-	
25 years	Atopic eczema (n=23)	0.87 (0.65-1.18)	0.371	0.80 (0.57-1.12)	0.200	104
	Non-atopic eczema (n=8)	1.04 (0.70-1.54)	0.862	1.18 (0.77-1.83)	0.449	
	Asymptomatic sensitized (n=35)	0.90 (0.70-1.17)	0.426	0.97 (0.73-1.29)	0.844	
	Asymptomatic (n=38)	Reference group	-	Reference group	-	

† Adjusted for the mother's age when gave birth, the presence of siblings, maternal prenatal smoking, dog ownership, maternal atopy history, maternal education, and paternal education.

‡ N: The total number of participants with vitamin D and eczema outcomes. n: of these, the number in each eczema/sensitisation subclass.

§ MOR: Multinominal OR. aMOR: Adjusted multinominal OR.
